# Fruit and Vegetable Consumption and Sarcopenia among Older Adults in Low- and Middle-Income Countries

**DOI:** 10.3390/nu12030706

**Published:** 2020-03-06

**Authors:** Ai Koyanagi, Nicola Veronese, Marco Solmi, Hans Oh, Jae Il Shin, Louis Jacob, Lin Yang, Josep Maria Haro, Lee Smith

**Affiliations:** 1 Research and Development Unit, Parc Sanitari Sant Joan de Déu, CIBERSAM, 08830 Barcelona, Spain; louis.jacob.contacts@gmail.com (L.J.); jmharo@pssjd.org (J.M.H.); 2 ICREA, Pg. Lluis Companys 23, 08010 Barcelona, Spain; 3 Department of Internal Medicine and Geriatrics, University of Palermo, 90133 Palermo, Italy; ilmannato@gmail.com; 4 Department of Neurosciences, University of Padova, 90133 Padova, Italy; marco.solmi83@gmail.com; 5 Padova Neuroscience Center, University of Padova, 90133 Padova, Italy; 6 Suzanne Dworak Peck School of Social Work, University of Southern California, Los Angeles, CA 90007, USA; hansoh@gmail.com; 7 Department of Pediatrics, Yonsei University College of Medicine, Seoul 03372, Korea; SHINJI@yuhs.ac; 8 Faculty of Medicine, University of Versailles Saint-Quentin-en-Yvelines, 78000 Versailles, France; 9 Department of Cancer Epidemiology and Prevention Research, Cancer Control Alberta, Alberta Health Services, Calgary, AB T2S 3C3, Canada; linyang33@gmail.com; 10 Departments of Oncology and Community Health Sciences, University of Calgary, Calgary, AB T2N 1N4, Canada; 11 The Cambridge Centre for Sport and Exercise Sciences, Anglia Ruskin University, Cambridge CB1 1PT, UK; Lee.Smith@anglia.ac.uk

**Keywords:** sarcopenia, fruit, vegetable, older people, low- and middle-income countries

## Abstract

Fruit and vegetable consumption may protect against sarcopenia but there are no studies on this topic from low- and middle-income countries (LMICs). Thus, we assessed this association among older adults from six LMICs. Community-based cross-sectional data of the Study on Global Aging and Adult Health were analyzed. Sarcopenia was defined as the presence of low skeletal muscle mass based on indirect population formula, and either slow gait or low handgrip strength. Quintiles of vegetable and fruit consumption were created based on the number of servings consumed on a typical day. Multivariable logistic regression analysis was conducted. The sample consisted of 14,585 individuals aged ≥65 years (mean (SD) age 72.6 (11.4) years; 55% females). Adjusted analyses showed that overall, compared to the lowest quintile (Q1), the highest quintile (Q5) of fruit consumption was associated with a 40% lower odds for sarcopenia (OR = 0.60; 95% CI = 0.42−0.84) but this association was largely driven by the strong association among females (e.g., Q5 vs. Q1 OR = 0.42; 95% CI = 0.24−0.73), with no significant associations found among males. Vegetable consumption was not significantly associated with sarcopenia. Future studies of longitudinal design may shed light on whether increasing fruit consumption among older females in LMICs may reduce risk for sarcopenia.

## 1. Introduction

Sarcopenia is an age-related disease characterized by a progressive loss of muscle mass and function, and is one of the most prominent physiological changes associated with aging [1]. For example, between age 40 and 80 years, total skeletal muscle mass declines by 30%–50% [2]. Multifactorial environmental and genetic factors [3] (e.g., low grade chronic inflammation, hormonal changes, oxidative stress, inactivity, diet, age-related chronic diseases) are likely to be implicated in the pathogenesis of sarcopenia [4]. Sarcopenia is highly prevalent in the general population with its prevalence among people aged ≥60 years estimated to be around 10% [5]. Given that the number of people aged ≥65 years is expected to nearly double between 2019 and 2050, from 703 million to 1.5 billion, while the proportion is projected to increase from 9% to 16% [6], the prevalence of sarcopenia will increase drastically in the coming years. Identifying the risk factors for sarcopenia is important as it is associated with a variety of adverse health outcomes such as falls, fractures, dependency, use of hospital services, institutionalization, poor quality of life, as well as higher mortality [7,8]. Furthermore, there are currently no effective pharmacological treatments for sarcopenia. Thus, it is imperative to identify potentially modifiable risk factors for this condition.

Currently, there is growing interest in the role of dietary behavior on the development of sarcopenia [4,9,10,11,12]. However, to date, there are only a few studies on the association between fruit and vegetable consumption and sarcopenia. Fruits and vegetables, which are rich in vitamins (e.g., vitamin C and A), minerals (e.g., electrolytes), phytochemicals (e.g., antioxidants) and dietary fiber [13], are regarded as essential components of a healthy diet, and their consumption has been linked with reduced risk for cancer, cardiovascular diseases, and premature mortality [14]. It is possible that the antioxidants contained in fruits and vegetables may defend against the negative effects of oxidative stress and lead to lower risk for sarcopenia, as oxidative stress has been shown to play a role in the genesis of sarcopenia [15]. Specifically, increased generation of reactive oxygen species and reduction in antioxidants with age could lead to increasing proteolysis and decreasing muscle protein synthesis, leading to reduction in muscle mass [15]. Furthermore, the alkaline-forming property of a diet rich in vegetables and fruits may potentially reduce protein degradation in the muscle [16]. Indeed, one cross-sectional Korean study among older adults (823 men and 1089 women aged ≥65 years) found that sarcopenia is more common in men and women who consume less fruits, and also men who consume less vegetables [17]. Furthermore, one study from Hong Kong (*n* = 3957) found that the dietary pattern “vegetables–fruits” was associated with lower odds for sarcopenia in men aged ≥65 years cross-sectionally [18]. Finally, a small randomized controlled trial conducted in the UK found a trend towards greater increase in grip strength in the group that consumed higher portions of fruits and vegetables [19]. 

However, these studies were conducted in high-income settings, and the lack of studies from low- and middle-income countries (LMICs) is a major omission as population ageing is occurring more rapidly in LMICs than in high-income countries, with more than two-thirds of older people projected to be living in LMICs in 2050 [6]. Furthermore, the proportion of people consuming adequate levels of fruit and vegetables has been reported to be very low in this setting possibly due to limited availability and high prices [20,21]. Thus, the aim of the study was to examine the association of fruit and vegetable consumption with sarcopenia using nationally representative data from six LMICs.

## 2. Materials and Methods

### 2.1. The Survey

Data from the Study on Global Ageing and Adult Health (SAGE) were analyzed. These data are publicly available through http://www.who.int/healthinfo/sage/en/. This survey was undertaken in China, Ghana, India, Mexico, Russia, and South Africa between 2007 and 2010. Based on the World Bank classification at the time of the survey, Ghana was the only low-income country, and China and India were lower middle-income countries although China became an upper middle-income country in 2010. The remaining countries were upper middle-income countries. 

Details of the survey methodology have been published elsewhere [22]. Briefly, in order to obtain nationally representative samples, a multistage clustered sampling design method was used. The sample consisted of adults aged ≥18 years with oversampling of those aged ≥50 years. Trained interviewers conducted face-to-face interviews using a standard questionnaire. Standard translation procedures were undertaken to ensure comparability between countries. The survey response rates were: China 93%; Ghana 81%; India 68%; Mexico 53%; Russia 83%; and South Africa 75%. Sampling weights were constructed to adjust for the population structure as reported by the United Nations Statistical Division. Ethical approval was obtained from the WHO Ethical Review Committee and local ethics research review boards. Written informed consent was obtained from all participants.

### 2.2. Sarcopenia

Following the criteria used in a previous publication using the same dataset [23], sarcopenia was defined as having low skeletal muscle mass (SMM) as reflected by lower skeletal mass index (SMI) and either a slow gait speed or a weak handgrip strength [24]. Skeletal muscle mass (SMM) was calculated as the appendicular skeletal muscle mass (ASM) based on the equation proposed by Lee and colleagues: ASM = 0.244*weight + 7.8*height + 6.6*sex − 0.098*age + race − 3.3 (where female = 0 and male = 1; race = 0 (White and Hispanic), race = 1.9 (Black) and race = −1.6 (Asian)) [25]. ASM was further divided by BMI based on measured weight and height to create a skeletal muscle mass index (SMI) [26]. Low SMM was defined as the lowest quintile of the SMI based on sex-stratified values. The higher range of SMI for the lowest quintile of the SMI was 1.04. Country-specific cut-offs were only used to determine low SMI, as this indicator is likely to be affected by racial differences in body composition. Gait speed was based on a 4 m timed walk and was measured by asking the participant to walk at a rapid pace, as fast as he/she safely can. The interviewer recorded the time to completion of the 4 m walk. Slow gait speed referred to the lowest quintile of walking speed based on height, age, and sex-stratified values [27,28]. Weak handgrip strength was defined as <30 kg for men and <20 kg for women using the average value of the two handgrip measurements of the dominant hand [29]. 

### 2.3. Fruit and Vegetable Consumption

Participants were asked the two following questions: “How many servings of fruit do you eat on a typical day?” and “How many servings of vegetables do you eat on a typical day?” The participants were grouped into five categories based on quintiles of the answer to these questions [17]. 

### 2.4. Covariates

The selection of the covariates was based on previous literature [17] and included sex, age, highest education achieved (primary, secondary, tertiary), wealth quintiles based on country-specific income, physical activity, current smoking, current drinking (alcohol use in the past 30 days), body mass index (BMI) based on measured weight and height (<18.5, 18.5–24.9, 25.0–29.9, ≥30.0 kg/m^2^), and chronic physical conditions. Levels of physical activity were assessed with the Global Physical Activity Questionnaire and were classified as low, moderate, and high based on conventional cut-offs [30]. We included all 10 chronic physical conditions (angina pectoris, arthritis, asthma, cataract, chronic lung disease, diabetes mellitus, edentulism, hearing problems, hypertension, stroke), assessed according to self-report of diagnosis, symptoms, interviewer observation, and blood pressure measurement (see Supplementary Materials Table S1 for details), for which data were available in SAGE. The total number of chronic conditions was summed for each individual. 

### 2.5. Statistical Analysis

The analysis was restricted to those aged 65 years or older because sarcopenia is an age-related condition. The difference in sample characteristics by sarcopenia was tested by Chi-squared tests for categorical variables and Student’s *t*-tests for continuous variables. Multivariable logistic regression analysis was conducted to assess the association between fruit or vegetable consumption based on quintiles (exposure) and sarcopenia (outcome) using the overall sample and sex-stratified samples. Fruits and vegetables were analyzed separately as a previous study showed that the effects of fruits and vegetables on sarcopenia risk may differ [17]. For example, fruits contain a higher level of fructose as compared to vegetables, and a high-fructose diet intake may lead to modifications in muscle function [31,32]. The analysis was adjusted for sex, age, education wealth, physical activity, smoking, alcohol consumption, BMI, number of chronic conditions, and country with the exception of the sex-stratified analysis, which was not adjusted for sex. Furthermore, the analysis on fruit consumption was adjusted for vegetable consumption, and the analysis on vegetable consumption was adjusted for fruit consumption. 

Adjustment for country was done by including dummy variables for each country in the model as in previous SAGE publications [33,34]. All variables were included in the models as categorical variables with the exception of age and number of chronic conditions (continuous variables). The sample weighting and the complex study design were taken into account in all analyses. Results from the regression analyses are presented as odds ratios (ORs) with 95% confidence intervals (CIs). The level of statistical significance was set at *p* < 0.05. 

## 3. Results

A total of 14,585 participants aged ≥65 years were included in the analysis (China *n* = 5360; Ghana *n* = 1975; India *n* = 2441; Mexico *n* = 1375; Russia *n* = 1950; South Africa *n* = 1484). The mean age was 72.6 (SD, 11.4) and 55% were females. The number of servings of fruits per day in each quintile were: Q1 = 0 servings; Q2 = 1 serving; Q3 = 2 servings; Q4 = 3 servings; Q5 = 4 + servings. The corresponding figures for vegetables were 0–1, 2, 3, 4–6, and 7+, respectively. The prevalence of sarcopenia was 15.7%. Those with sarcopenia were significantly more likely to be older, have lower levels of education, wealth, and physical activity, while they were also more like to smoke and consume alcohol, and have greater number of chronic conditions (Table 1).

**Figure 1:**
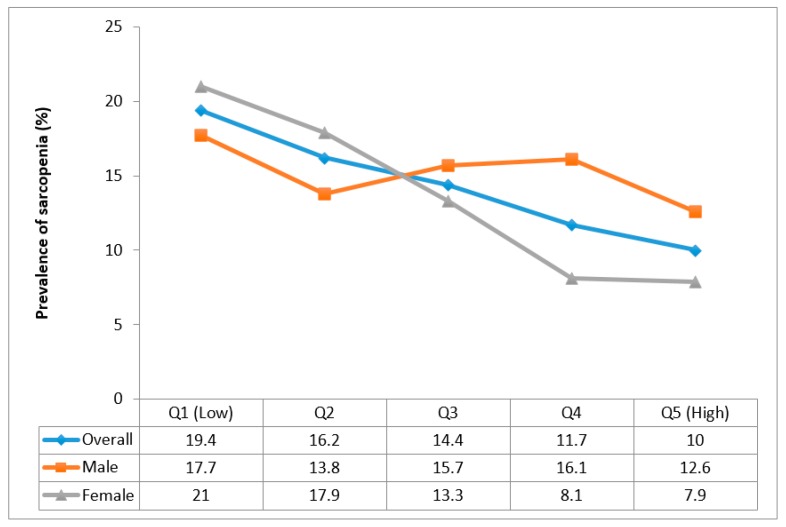
Prevalence of sarcopenia by quintiles of fruit consumption.

There was an overall trend for increasing fruit consumption to be associated with lower prevalence of sarcopenia but this was largely driven by the trend among females (Figure 1). Specifically, among females, the prevalence of sarcopenia decreased from 21% (lowest quintile Q1) to 7.9% (highest quintile Q5). 

**Figure 2:**
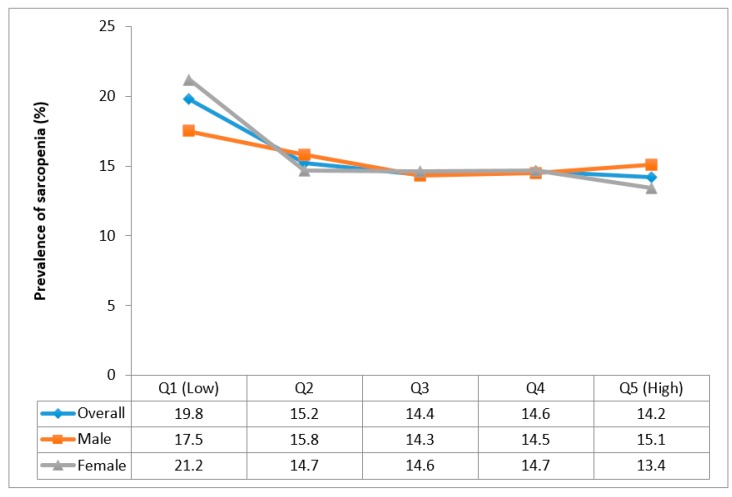
Prevalence of sarcopenia by quintiles of vegetable consumption.

For vegetable consumption, only a slight drop in the prevalence of sarcopenia was observed between Q1 and Q2 with the prevalence between Q2 and Q5 being almost equal in the overall and sex-stratified samples (Figure 2). 

In the adjusted analysis, overall, compared to the lowest quintile (Q1), Q3, Q4, and Q5 were associated with significantly lower odds for sarcopenia (e.g., Q5 vs. Q1 OR = 0.60; 95%CI = 0.42–0.84) (Table 2). This association was particularly pronounced among females but was not significant for any of the quintiles for males. There were no significant associations for vegetable consumption in the overall sample or sex-stratified samples.

## 4. Discussion

In our study, we found that fruit consumption is overall significantly associated with lower odds for sarcopenia among adults aged ≥65 years in LMICs, but that this association was only found among females. Specifically, compared to women in the lowest levels of fruit consumption (Q1), those in Q4 and Q5 had an approximately 60% lower odds for sarcopenia. Vegetable consumption was not associated with sarcopenia in our study. The strengths of the study include the large sample size and the use of nationally representative datasets from six LMICs which comprise nearly half of the worldwide population [22]. To the best of our knowledge, our study is the largest study on this topic to date, while it is also the first study from LMICs.

The inverse association between fruit consumption and sarcopenia observed in our study may be explained by the effects of antioxidant nutrients such as vitamin C and carotenoids [35], which are found in abundance in fruits [13]. The mechanisms of aging of skeletal muscle are related with factors such as increases in circulating cytokines (e.g., interleukin-6, tumour necrosis factor-α, interleukin-10, interleukin-15) [36] and production of reactive oxygen species, which can cause direct cellular damage of skeletal muscle fibers and DNA, and also have negative effects on synthesis of protein [37]. It is possible that exogenous antioxidant vitamins have potential importance for maintaining skeletal muscle mass especially in old age as endogenous antioxidant efficiency is reduced as people age [38]. For example, lower plasma vitamin C concentration was associated with poorer physical performance among elderly Japanese women [39], while greater dietary intakes of most antioxidants (e.g., vitamin C and beta-carotene) were associated with higher skeletal muscular strength among elderly people in an Italian study [40]. Additionally, the alkaline-forming properties of fruits may also act as a buffer against oxidative stress and inflammation, and increase skeletal muscle mass [41].

However, in our study, a significant inverse association between fruit consumption and sarcopenia was only found among women and not men. Although our findings among women were similar to the Korean study, our results are in contrast to this previous study that found similar associations among both men and women [17]. Specifically, in this Korean study, the ORs (95% CIs) when those in the highest quintile of fruit consumption were compared to the lowest were 0.30 (0.13–0.70) for men and 0.39 (0.18–0.83) for women. Although the reason why no significant associations were found in men in our study is unknown, it is possible that women in our study were consuming fruits that are more nutritious than men as one study showed that the consumption of fruits and vegetables among women may be motivated more by the greater knowledge on the nutritional benefits of fruits or vegetables than men [42]. Additionally, the etiology of sarcopenia may differ between men and women mainly due to differences in hormone profiles [43,44,45]. Furthermore, one study found that muscle of older women can display higher heterogeneity in myofiber size and phenotype composition compared to older men [46]. Some studies have reported that well-established risk factors for sarcopenia such as physical activity may be more important in men than women [47]. Thus, it is possible that nutrition may be a less important factor for the development of sarcopenia among men than women at least in some settings although this would require further investigation. 

Finally, we found no association between vegetable consumption and sarcopenia for both sexes. In the Korean study, although vegetable consumption was not associated with sarcopenia among women, this was statistically significant for men [17]. The reasons for the discrepant results may be attributable to factors such as different methodology (e.g., definition of sarcopenia), or the setting in which the studies were conducted. Specifically, the types of vegetables consumed by different populations can vary substantially. Furthermore, for example, antioxidant values of any food component may differ widely for a variety of reasons (e.g., growing conditions, storage conditions, cooking process) [48]. Thus, studies from a variety of settings are necessary to assess the degree to which findings are context-specific. 

The results of this study should be interpreted in the light of several limitations. First, most of the data were based on self-report, which is subject to bias (e.g., recall, social desirability bias). Second, ASM was based on a population equation and not direct assessment. However, this has been validated against gold standard methods such as magnetic resonance imaging and dual-energy X-ray absorptiometry in diverse populations, and good concordance rates have been reported [25,49]. Furthermore, these methods are costly and often impractical for population-based surveys. Third, it is also possible that people who consume greater amounts of fruits and vegetables are those who are more health-conscious and consume other types of food that may promote health. We were unable to adjust for other types of food due to lack of data but this may not have been a major limitation as for example, previous studies have shown that protein intake, which reduces risk for sarcopenia [50], is not associated with increased fruit/vegetable consumption [51]. Furthermore, a study from Korea found that the significant inverse association between fruit or vegetable consumption and sarcopenia remained even after adjusting for other dietary factors [17]. Next, for sarcopenia, there are still no universal operative definitions. Our definition was based on that proposed by international groups of experts, but it is possible that the results may differ if a different definition for sarcopenia was used. Finally, given the cross-sectional nature of the study, causality and temporal associations cannot be established. 

In conclusion, the present study found that fruit consumption is associated with lower odds for sarcopenia among women aged ≥65 years living in six LMICs. Further studies from a variety of settings are necessary to understand whether the association is context-specific, and whether contradicting results found in previous studies can be explained by factors such as differences in the predominant types of vegetable and fruits consumed. In addition, future studies are needed to understand the basis of sex-differences that has been observed in our study and previous studies, and why only fruit consumption was associated with sarcopenia in our study despite the fact that vegetables also consist of nutritional components that may theoretically delay the onset of sarcopenia (e.g., antioxidant nutrients). Finally, confirmatory studies of longitudinal design are necessary to examine whether fruit intake especially among women may lead to reduction in sarcopenia among older people living in LMICs.

## Figures and Tables

**Table 1:** Sample characteristics (overall and by sarcopenia).

			Sarcopenia		
		Overall	No	Yes	*p*-Value ^a^
Sex	Female	55.0	54.5	55.1	<0.769
	Male	45.0	45.5	44.9	
Age	Mean (SD)	72.6 (11.5)	71.4 (10.1)	76.1 (12.4)	<0.001
Education	Primary	63.7	64.8	77.5	<0.001
	Secondary	29.9	28.9	19.2	
	Tertiary	6.4	6.2	3.4	
Wealth	Poorest	21.7	19.8	32.1	<0.001
	Poorer	21.0	20.1	22.5	
	Middle	20.4	20.4	17.9	
	Richer	17.5	18.9	15.0	
	Richest	19.4	20.9	12.6	
Physical activity	High	35.2	38.6	29.0	<0.001
	Moderate	25.2	26.1	25.6	
	Low	39.6	35.3	45.4	
Smoking	No	70.7	68.9	69.9	0.705
	Yes	29.3	31.1	30.1	
Alcohol consumption	No	86.1	85.2	89.9	0.001
	Yes	13.9	14.8	10.1	
BMI (kg/m^2^)	<18.5	19.3	20.1	20.2	0.061
	18.5–24.9	46.4	47.3	45.1	
	25.0–29.9	23.9	23.7	21.8	
	≥30	10.4	9.0	12.9	
No. of chronic conditions	Mean (SD)	2.4 (2.9)	2.3 (2.9)	2.6 (2.9)	<0.001

Abbreviation: SD—standard deviation; BMI—body mass index. Data are percentage unless otherwise stated. ^a^ *p*-value was calculated by Chi-squared tests for categorical variables and Student’s *t*-tests for continuous variables.

**Table 2:** Association of fruit and vegetable consumption (quintiles) with sarcopenia estimated by multivariable logistic regression.

		Overall		Male		Female	
		OR	95%CI	OR	95%CI	OR	95%CI
Fruit consumption ^a^	Q1 (Low)	1.00		1.00		1.00	
	Q2	0.85	(0.62, 1.16)	0.74	(0.50, 1.10)	0.87	(0.55, 1.38)
	Q3	0.74 *	(0.55, 1.00)	0.86	(0.58, 1.28)	0.56 **	(0.37, 0.83)
	Q4	0.64 *	(0.43, 0.95)	1.12	(0.71, 1.77)	0.38 **	(0.21, 0.69)
	Q5 (High)	0.60 **	(0.42, 0.84)	0.81	(0.53, 1.25)	0.42 **	(0.24, 0.73)
Vegetable consumption ^b^	Q1 (Low)	1.00		1.00		1.00	
	Q2	0.85	(0.63, 1.15)	0.96	(0.57, 1.63)	0.86	(0.53, 1.38)
	Q3	0.91	(0.60, 1.38)	0.95	(0.52, 1.74)	0.97	(0.51, 1.85)
	Q4	0.89	(0.59, 1.33)	0.81	(0.41, 1.56)	1.09	(0.61, 1.95)
	Q5 (High)	0.99	(0.62, 1.58)	1.00	(0.49, 2.03)	1.07	(0.55, 2.09)

Abbreviation: OR—odds ratio; CI—confidence interval. ^a^ Models are adjusted for sex, age, education wealth, physical activity, smoking, alcohol consumption, BMI, number of chronic conditions, vegetable consumption, and country with the exception of the sex-stratified analysis which was not adjusted for sex. ^b^ Models are adjusted for sex, age, education wealth, physical activity, smoking, alcohol consumption, BMI, number of chronic conditions, fruit consumption, and country with the exception of the sex-stratified analysis which was not adjusted for sex. * *p* < 0.05, ** *p* < 0.01.

## Supplementary Materials

The following are available online at https://www.mdpi.com/2072-6643/12/3/706/s1, Table S1: Details on the diagnosis of chronic conditions.
