# Supplementary material for: Fruit and Vegetable Consumption and Sarcopenia among Older Adults in Low- and Middle-Income Countries
Source: Nutrients. 2020 Mar 6;12(3):706. doi: 10.3390/nu12030706 (PMC7146581; doi:10.3390/nu12030706)
Supplement: Supplementary file 1 [file nutrients-12-00706-s001.pdf]

Supplementary material

Table S1 Details on the diagnosis of chronic conditions

Condition	(a) Self-reported diagnosis	(b) Symptom-based algorithm or other method of diagnosis[a]
Angina	Have you ever been diagnosed with angina or angina pectoris (a heart disease)?	Rose questionnaire[1]
Arthritis	Have you ever been diagnosed with/told you have arthritis (a disease of the joints, or by other names rheumatism or osteoarthritis)?	Affirmative answers to all four of the following:<br>1. During the last 12 months, have you experienced pain, aching, stiffness or swelling in or around the joints (e.g., in arms, hands, legs or feet) which were not related to an injury and lasted for more than a month?<br>2. During the last 12 months, have you experienced stiffness in the joint in the morning after getting up from bed, or after a long rest of the joint without movement?<br>3. Did this stiffness last for less than 30 minutes?<br>4. Did this stiffness go away after exercise or movement in the joint?
Asthma	Have you ever been diagnosed with asthma (an allergic respiratory disease)?	1. During the last 12 months, have you experienced attacks of wheezing or whistling breathing? (Yes)<br>**AND**<br>2. "Yes" to at least one of the following (past 12 months):<br>(a) Have you experienced an attack of wheezing that came on after you stopped exercising or some other physical activity?<br>(b) Have you had a feeling of tightness in your chest?<br>(c) Have you woken up with a feeling of tightness in your chest in the morning or any other time?<br>(d) Have you had an attack of shortness of breath that came on without an obvious cause when you were not exercising or doing some physical activity?
Cataract	In the last 5 years, were you diagnosed with a cataract in one or both of your eyes (a cloudiness in the lens of the eye)?	1. In the last 12 months, have you experienced cloudy or blurry vision? (Yes)<br>**AND**<br>2. In the last 12 months, have you experienced vision problems with light, such as glare from bright lights, or halos around lights? (Yes)

Chronic lung disease	Have you ever been diagnosed with chronic lung disease (emphysema, bronchitis, COPD)?	1. During the last 12 months, have you experienced any shortness of breath at rest (while awake)?<br>(Yes)<br>**OR**<br>2. "Yes" to both of the following (past 12 months):<br>(a) Have you experienced any coughing or wheezing for 10 minutes or more at a time?<br>(b) Have you experienced any coughing up of sputum or phlegm on most days of the month for at least 3 months?
Diabetes	Have you ever been diagnosed with diabetes (high blood sugar)? (not including diabetes associated with a pregnancy)	NA
Edentulism	"Have you lost all of your natural teeth?"	NA
Hearing problem	NA	Interviewer observation
Hypertension	Have you ever been diagnosed with high blood pressure (hypertension)?	Blood pressure was measured three times with a one-minute interval with the use of a wrist blood pressure monitor (Medistar Wrist Blood Pressure Model S) and the mean value of the three measurements was calculated. Hypertension was defined as having at least one of the following: systolic blood pressure ≥140 mmHg; diastolic blood pressure ≥90 mmHg.
Stroke	Have you ever been told by a health professional that you have had a stroke?	NA

For all chronic conditions, we assumed that the individual had the condition if they fulfilled at least one of the following: (a) affirmative answer to self-reported diagnosis or (b) symptom-based algorithm or other method of diagnosis.

[a] These algorithms have been used in previous publications [2,3] and those of arthritis, asthma, and chronic lung disease have been validated.[2,4]
